# Using pedicle flaps for donor-site closure after harvesting subscapular artery flap in pediatric patients

**DOI:** 10.1016/j.jpra.2026.07.013

**Published:** 2026-07-17

**Authors:** Qifeng Ou, Ziyue Wang, Panfeng Wu, Juyu Tang

**Affiliations:** a Department of Orthopaedics, Hand and Microsurgery, Xiangya Hospital, Central South University, Changsha, Hunan, 410008, China; b School of Nursing and Midwifery, University of Galway, Galway, Ireland; c National Clinical Research Center for Geriatric Diseases (XiangYa Hospital), Changsha City, Hunan, 410008, China

**Keywords:** Donor site closure, Flap, Subscapular artery perforator flap, Pediatric patients

## Abstract

**Background:**

Flaps based on the subscapular artery are versatile options in reconstructive surgery. However, after extensive flap harvest, primary donor-site closure may be difficult, and skin grafting may be required. In pediatric patients, skin grafting may be associated with additional donor-site morbidity, scar contracture, contour mismatch, and potential growth-related concerns. Although local pedicled flaps have been used for donor-site closure in regions such as the anterolateral thigh and forearm, their application after subscapular artery system flap harvest remains limited.

**Methods:**

We report three pediatric patients aged 4–7 years who underwent reconstruction with subscapular artery flaps for complex defects of the foot dorsum, upper arm, and heel. In each case, the subscapular donor-site defect could not be closed primarily. Instead of skin grafting, individualized local pedicled flaps, including triangular perforator-based, transposition, and keystone-like trapezoidal designs, were used to achieve tension-free closure. Flap design was based on donor-site geometry, adjacent tissue laxity, and available perforators.

**Results:**

All subscapular artery flaps and local pedicled flaps survived completely. Donor-site defects were closed without skin grafting, wound dehiscence, or delayed healing. The donor sites healed with linear scars, and no donor-site complications were recorded during follow-up of 2 months to 3 years.

**Conclusion:**

In selected pediatric patients, individualized local pedicled flaps may provide a feasible option for closing subscapular donor-site defects when primary closure is not possible. This approach may reduce closure tension and avoid skin grafting, but larger studies with longer follow-up are needed to better define its indications and long-term outcomes.

## Introduction

Flaps based on the subscapular artery system offer remarkable versatility in reconstructive surgery.^1^ They can be configured in multiple forms, including fasciocutaneous, musculocutaneous, and composite designs, and are widely used for reconstruction of complex defects in the extremities and craniofacial region.

While primary donor-site closure remains ideal, larger flap harvests often preclude direct approximation.^2^ Forced closure under tension risks wound dehiscence and delayed healing. Skin grafting, though an alternative, introduces a secondary donor site and presents challenges such as colour mismatch and muscle adhesion, which may compromise scapular function.^3^^,^^4^ These concerns are particularly relevant in pediatric patients, in whom grafted skin has less elasticity than local tissue and may interfere with symmetric growth of the thoracodorsal region.

The subscapular region provides a relatively broad and mobile skin–soft tissue envelope over the posterior thorax, with adjacent tissue laxity that can often be recruited for local flap advancement or transposition.^5^ In addition, reliable regional perforators around the subscapular donor site provide an anatomical basis for designing local pedicled flaps to achieve closure without skin grafting in selected cases.^6^^,^^7^

Local pedicled flaps have proven effective for donor-site closure following harvest of established workhorse flaps like the anterolateral thigh flap.^3^ However, literature describing their use after subscapular artery flap harvest remains scarce.

This study presents three cases where customized local pedicled flaps successfully achieved tension-free closure of subscapular donor-site defect. By demonstrating the adaptability and reliability of this approach, we provide valuable insights that may guide surgical strategy in similar challenging scenarios.

### Surgical approach

In three pediatric patients, the donor-site defects in the thoracodorsal region could not be closed primarily after harvest of subscapular artery flaps for coverage of extremity wounds. In these cases, local advancement of perforator flap or transposition flaps were designed around the thoracodorsal donor-site defect to achieve wound closure.

The design of these local flaps involved three steps. First, the laxity of the tissue surrounding the thoracodorsal defect was assessed using a pinch test. Second, perforator vessels around the defect were localized preoperatively using a handheld Doppler ultrasound device and marked on the skin. Third, the flap was designed according to the size and shape of the defect. In general, either a triangular flap or a trapezoid flap, similar to a keystone flap, was designed. For triangular flaps, the flap was placed on the side of the defect with greater skin laxity. Based on the perforator identified preoperatively, the flap base was positioned along one edge of the defect, and the line connecting the center of the defect and the marked perforator was used as the flap axis. The apical angle of the flap was maintained at 30°–45° For trapezoid flaps, the flap was also designed on the side with greater skin laxity. One edge of the defect served as the short base of the trapezoid, while the long base was located on the opposite side of the flap. The flap width was designed to be greater than the width of the defect, and the two basal angles of the trapezoid were maintained at 30°–60°.

During flap advancement, short perforators that restricted flap mobilization were divided and ligated. In general, 1–3 relatively large and long perforators were preserved to maintain perfusion to the entire flap, including its distal margins. Excessive skeletonization of the perforators was avoided; dissection was limited to the extent required to allow sufficient flap advancement for tension-free wound coverage. After flap inset, a silicone half-tube drain was placed. The reconstructed extremity was immobilized for 5–7 days to minimize excessive movement, shear force, or tension that could compromise the flap pedicle or vascular anastomosis. During the first 2 postoperative weeks, the child was positioned to avoid direct compression of the flap donor site, usually in the lateral decubitus position. Sutures were removed 14 days postoperatively. During follow-up, flap survival, flap texture, donor-site condition, wound healing, and wound-related complications were recorded.

## Case presentation

### Case one

A 4-year-old boy presented with a soft-tissue defect on the dorsum of his right foot following a traffic accident, which involved tendon exposure and necessitated flap coverage. A free double-paddle perforator flap based on the subscapular artery system was designed (with perforators preoperatively mapped by doppler). The two paddles (measuring 12 × 7 cm and 11 × 6 cm), each originating from separate perforators but joined at a common bifurcation point, were harvested from the right subscapular region. These paddles were inset side-by-side to resurface the dorsal foot defect. Microvascular anastomosis was performed end-to-end to the dorsal artery of the foot (Supplementary Figure 1).

**Fig. 1:**
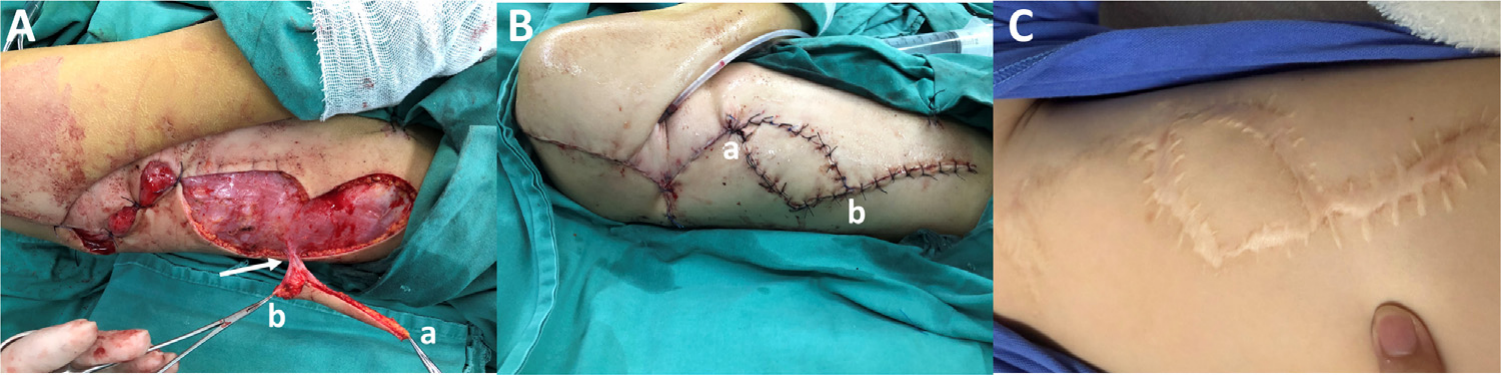
Case 1. Donor-site closure in the subscapular region using a perforator-based triangular propeller flap. (A) Donor-site defect before local flap transfer. (B) Flap inset after advancement and approximately 180° rotation of the perforator-based triangular flap. (C) Flap survival and donor-site appearance 3 years after surgery. The white arrow indicates the perforator. Labels a and b indicate the distal and proximal corners of the triangular flap, respectively.

The donor site was not amenable to primary closure due to excessive tension, particularly in the mid-portion of the defect. A small local perforator-based advancement triangular flap was therefore raised from the adjacent low-tension area along the inferior border of the defect. The flap was based on one perforator (Fig. 1A), rotated 180 degrees, allowing its distal portion to cover the high-tension region. To allow adequate upward mobilization of the flap, the perforator was partially skeletonized over an appropriate length, with a small cuff of surrounding fascial tissue preserved around the pedicle. The donor site for this perforator-based advancement triangular flap was closed primarily, without the need for skin grafting (Fig. 1).

The postoperative course was uneventful. Both the free flap and the donor site transposition flap survived completely. The wounds on the foot and the back healed well, resulting only in linear scars. At the 3‑year follow‑up, no complications in the thoracodorsal region were documented.

### Case two

A 4-year-old boy sustained a deep, extensive, and circular soft-tissue defect on the posterior aspect of the left upper arm due to a horse bite, which necessitated flap coverage. A pedicled flap (12 × 10 cm) was harvested from the subscapular region and transferred via a subcutaneous tunnel through the axilla to cover the arm defect (Supplementary Figure 2).

**Fig. 2:**
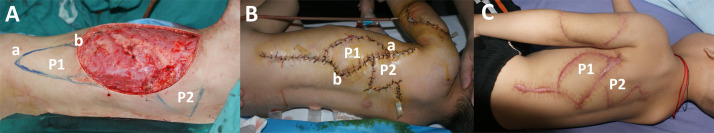
Case 2. Donor-site closure using two local flaps. (A) Design of two local flaps, labeled P1 and P2. (B) Immediate appearance after flap transfer for tension-free donor-site closure. (C) Flap survival and donor-site appearance 6 months after surgery. P1 indicates a perforator-based triangular flap designed inferior to the defect; labels a and b indicate the distal and proximal corners of this flap, respectively. P1 was advanced toward the defect and rotated approximately 180° to provide coverage. P2 indicates a transposition flap based on preserved soft-tissue continuity rather than an isolated perforator; it was mobilized and rotated laterally by approximately 40° to assist in defect closure.

Following flap elevation, the large donor site on the back could not be closed primarily. To achieve tension-free closure and avoid skin grafting, a two-flap technique was employed. First, an inferior pedicled triangular skin paddle, which would otherwise have been excised to avoid a dog-ear deformity, was preserved and designed as a perforator-based flap. This flap was advanced toward the donor-site defect and rotated approximately 180° to maximize defect coverage (Fig. 2B, P1). In addition, a second flap located medial to the donor-site defect was designed based on preserved soft-tissue continuity, without deliberate perforator dissection. This flap was mobilized and rotated laterally by approximately 40° to assist in closure of the remaining defect (Fig. 2B, P2).

The donor sites were ultimately closed by direct suture under minimal tension. The postoperative course was uneventful, with complete survival of all flaps—both the primary flap to the arm and the two local flaps used for donor site closure. All wounds healed satisfactorily, resulting in fine linear scars at both the recipient and donor sites. At the 6‑month follow‑up, no complications in the thoracodorsal region were documented.

### Case three

A 7-year-old girl presented with a deep soft-tissue defect over the posterior heel, sustained in a spoke-wheel injury, which resulted in exposure of the Achilles tendon. Definitive coverage with a pliable flap was required. A free bi-pedicle perforator flap (10.5 × 9 cm) based on the subscapular artery system was designed and harvested. After transfer, the flap was revascularized via anastomosis of its perforator vessel to the posterior tibial artery and its concomitant vein (Supplementary Figure 3).

**Fig. 3:**
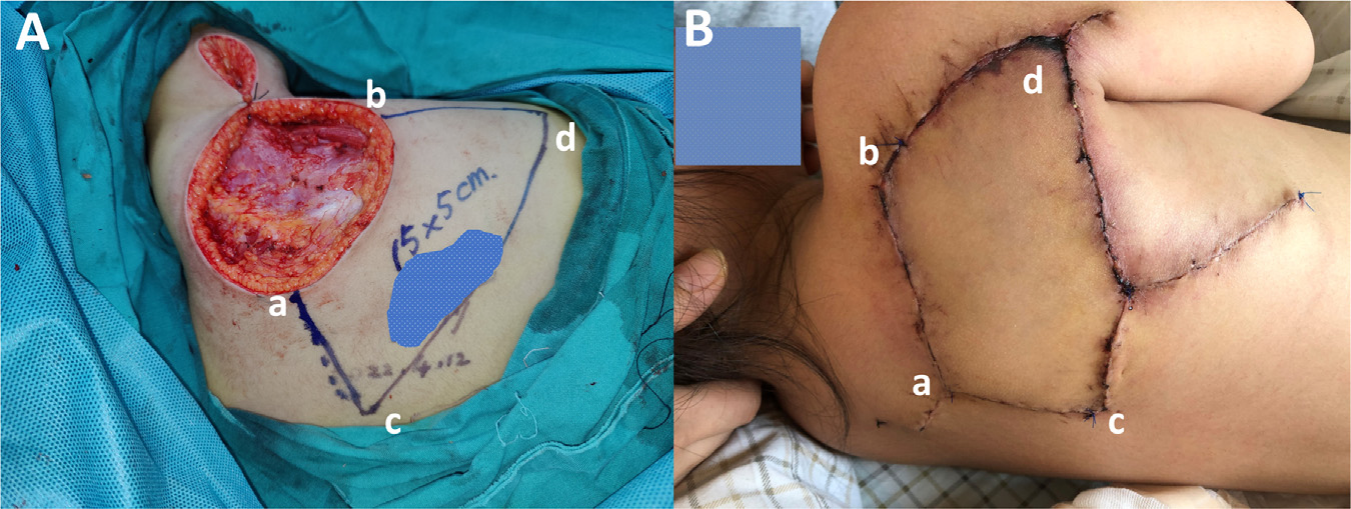
Case 3. Donor-site closure using a keystone-like trapezoidal flap. (A) Donor-site defect in the subscapular region and design of the keystone-like trapezoidal local flap. (B) Donor-site appearance 7 days after flap transfer. Labels a, b, c, and d indicate the four corners of the trapezoidal flap.

The donor site in the circumflex scapular region could not be closed primarily. To facilitate tension-free closure, a keystone-like trapezoidal advancement flap measuring 15 × 5 cm was designed inferior to the donor-site defect. The upper base of the trapezoid was placed along the margin of the donor-site defect, and the lower base was positioned in the area with greater skin laxity, as indicated by the pinch test. Intraoperatively, five perforators were identified. Three relatively large perforators that provided adequate perfusion to the flap were preserved, whereas two smaller and shorter perforators that limited flap advancement were divided and ligated. The flap was then advanced superiorly and rotated counterclockwise by approximately 40° to close the primary defect. The secondary defect resulting from the keystone flap transfer.^8^ was closed primarily, and no skin grafting was required (Fig. 3).

The postoperative course was uneventful, with complete survival of both the free circumflex scapular artery perforator flap and the keystone flap. The heel wound and the back donor site healed well, ultimately forming only fine linear scars. At the 2‑month follow‑up, no complications in the thoracodorsal region were documented.

## Discussion

Although limited to three cases, our experience suggests that individualized local pedicled flaps may be feasible for closing selected subscapular donor-site defects in pediatric patients when primary closure is not possible. In all three cases, adjacent lax tissue was recruited, with or without perforator-based mobilization, to reduce closure tension and avoid skin grafting.

When primary closure of a flap donor site is not feasible, skin grafting is commonly used.^9^ However, this option may be less desirable in children. Because pediatric patients continue to grow, grafted skin may have limited elasticity and may be associated with scar contracture, contour mismatch, adhesion to underlying tissues, and delayed wound healing.^9^ These problems may affect appearance, local soft-tissue development, shoulder or scapular-region mobility, and long-term donor-site quality. In our three cases, individualized local pedicled flaps allowed donor-site closure without skin grafting or delayed wound healing.

The use of local flaps for donor-site closure has been more widely described in other flap procedures, including anterolateral thigh.^10^ and radial forearm free flaps.^9^ where adjacent tissue transfer can reduce the need for skin grafting and improve donor-site healing. However, reports specifically addressing local flap closure after harvest of subscapular artery system flaps remain limited. Several strategies have been described to reduce donor-site tension and avoid skin grafting in this region. Multilobed latissimus dorsi flap designs can reduce the width of each skin paddle and facilitate primary donor-site closure.^11^ however, this strategy may still be insufficient when a large flap is required or when residual donor-site tension persists, as illustrated by Case 1 in the present study. Tissue expansion may increase available local tissue, but it requires staged treatment and is not suitable for acute traumatic defects.^12^ Local pedicled flaps therefore provide a practical alternative when direct closure remains difficult.

Reports specifically addressing local flap closure of donor-site defects after harvest of subscapular artery system flaps remain limited. Liu et al. described a freestyle pedicled thoracodorsal artery perforator flap to assist closure of a parascapular flap donor-site defect.^7^ More recently, Park et al. reported three cases in which dorsal scapular artery perforator rotational flaps were used to close donor defects after latissimus dorsi or thoracodorsal artery perforator flap harvest. Their design was relatively consistent, using longitudinally oriented flaps advanced inferiorly and rotated laterally by approximately 180°.^6^

The present cases differed from these previous reports in that the local flap design was individualized according to the size, location, residual laxity, and vascular basis of each donor-site defect. In Case 1, a double-paddle subscapular artery perforator flap reduced the overall donor-site width, leaving only an inferior high-tension area; therefore, a small triangular perforator-based flap was sufficient. In Case 2, the wider thoracodorsal artery-based flap created a larger lateral back defect, and two local flaps were used to recruit lax tissue from the inferior and medial margins. In Case 3, the donor-site defect was closer to the shoulder region after harvest of a circumflex scapular artery-based flap; a keystone-like trapezoidal flap was selected to simultaneously recruit the relatively lax inferior and medial skin. These cases suggest that donor-site closure after subscapular artery system flap harvest should not rely on a single fixed design but should be tailored to the defect geometry, surrounding tissue laxity, and available perforators.

This study has several limitations. First, only three pediatric cases were included, and the findings should therefore be interpreted as preliminary technical experience. Second, the flap designs were heterogeneous, which limits direct comparison among cases. Third, follow-up was limited, particularly for assessing scar maturation, growth-related donor-site changes, and long-term functional or aesthetic outcomes. Objective functional and scar assessments were not systematically performed. Larger studies with longer follow-up are needed to further define the indications and long-term value of this approach.

## Conclusion

In selected pediatric patients, individualized local pedicled flaps may provide a feasible option for closing subscapular donor-site defects when primary closure is not possible. By recruiting adjacent lax tissue, with or without perforator-based mobilization, this approach may reduce closure tension and avoid skin grafting. Larger studies with longer follow-up are needed to better define its indications and long-term donor-site outcomes.

## Author contribution

Qifeng Ou conceived the study, collected the data, designed the methodology, and drafted the initial manuscript. Ziyue Wang contributed to the conceptualization, manuscript drafting, and manuscript review. Panfeng Wu contributed to the surgical procedures and critically reviewed and revised the manuscript. Juyu Tang contributed to the surgical design, manuscript review, editing, and final approval. All authors read and approved the final version for submission.

## Declaration of generative AI in scientific writing

The authors used DeepSeek for language polishing during manuscript preparation. All content was verified and approved by the authors, who accept full responsibility for the published material.

## Ethical approval

The studies involving patients were reviewed and approved by Xiangya Hospital. Written informed consent was obtained from the individual(s) for the publication of any potentially identifiable images or data included in this article. The protocol was in accordance with the ethical standards of the Helsinki Declaration of 1975 and all subsequent revisions.

## Research registration

Not applicable. This study is a retrospective case series and not a clinical trial.

## Declaration of competing interest

The authors declare no potential conflicts of interest with respect to the research, authorship, and/or publication of this article.

## Data Availability

The data supporting the findings of this study are available from the corresponding author upon reasonable request.

## Appendix Supplementary materials

Image, application 1
