# Supplementary material for: Using pedicle flaps for donor-site closure after harvesting subscapular artery flap in pediatric patients
Source: JPRAS Open. 2026 Jul 17;51:504–10. doi: 10.1016/j.jpra.2026.07.013 (PMC13448422; doi:10.1016/j.jpra.2026.07.013)
Supplement: Supplementary file 1 [file mmc1.docx]

**Supplementary Figures**

**Supplementary Figure 1.** Intraoperative procedures of Case 1. (A–C) Free subscapular artery perforator flap designed as a double skin paddle (A), elevated (B), and used to resurface the soft-tissue defect on the foot dorsum (C). (D, E)

**Supplementary Figure 2.** Intraoperative planning and postoperative follow-up in Case 2. P0: the flap used to repair the upper arm defect; P1 and P2: local flaps used for donor-site closure. (A) Soft-tissue defect on the upper arm and the designed subscapular flap (P0). (B) The P0 flap after elevation. (C) The upper arm defect reconstructed with P0; the donor site could not be closed primarily. (D, E) Design and immediate transfer of local pedicled flaps (P1, P2) to achieve tension-free donor-site closure. (F) Six-month postoperative view.

**Supplementary Figure 3.** Case 3. (A–D) Reconstruction of the heel defect. (E, F) Donor-site closure using a keystone flap. (A) Design of the free bi-pedicle subscapular artery perforator flap. (B, C) Flap after elevation. (D) Inset of the flap to repair the posterior heel defect.
